# Efficacy and Safety of Opioid Analgesia in Acute Abdominal Presentations: A Systematic Review

**DOI:** 10.7759/cureus.117534

**Published:** 2026-10-06

**Authors:** Connor A Payne, Ganesh Prasad Neupane

**Affiliations:** 1 Emergency Medicine, Medical University of the Americas, Charlestown, KNA; 2 Pharmacology, Medical University of the Americas, Charlestown, KNA

**Keywords:** acute abdominal pain, analgesia, diagnostic accuracy, emergency department, non-opioid analgesia, opioid analgesia, pain management

## Abstract

Acute abdominal pain is a common emergency department presentation and is often associated with diagnostic uncertainty. Historically, opioid analgesia was used cautiously because of concern that it could alter physical examination findings and interfere with diagnostic accuracy. Non-opioid and opioid-sparing strategies are increasingly used for pain management. This systematic review evaluates the efficacy and safety of analgesic strategies for acute, non-traumatic abdominal pain, with attention to pain relief, adverse effects, rescue analgesia, and diagnostic accuracy. PubMed, Embase, and the Cochrane Central Register of Controlled Trials were searched for randomized comparative trials of opioid-containing treatment versus non-opioid, opioid-sparing, or placebo strategies. Eligible studies included adults aged 18 years or older with acute abdominal pain or an acute intra-abdominal cause of pain; studies limited to renal or ureteric colic were excluded. Of the 316 records identified, 45 duplicates were removed, 271 records were screened, 26 reports underwent full-text assessment, and 16 randomized trials were included. Across the included trials, opioids provided effective short-term analgesia but were not consistently superior to alternatives. Hydromorphone produced greater pain improvement than intravenous lidocaine at 90 minutes (between-group difference = 1.2 points, 95% confidence interval (CI) = 0.3-2.2) with less rescue analgesia. In acute pancreatitis, median Visual Analog Scale (VAS) reductions at 30 minutes were 45.5 (95% CI = 30-54) with tramadol, 41.5 (95% CI = 34-50) with paracetamol, and 40.5 (95% CI = 30-47) with dexketoprofen, with no statistically significant between-group difference. An erector spinae plane block produced lower pain scores and reduced rescue fentanyl compared with intravenous tramadol. In biliary colic, mean pain relief at 30 minutes was 3.8 (95% CI = 2.4-5.2) with ketorolac and 3.9 (95% CI = 2.4-5.3) with meperidine, with no statistically significant between-group difference. Butorphanol provided greater early pain relief than ketorolac at 15 minutes but not at 30 minutes. Low-dose morphine plus paracetamol did not significantly differ from standard-dose morphine, and intravenous lidocaine and morphine did not significantly differ at the primary 60-minute endpoint. Several placebo-controlled trials assessed diagnostic accuracy using different definitions and patient populations. In one trial, diagnostic accuracy was 86% with morphine and 85% with placebo (difference 1 percentage point, 95% CI = −11 to 12). This imprecise estimate does not rule out a clinically important difference, and the available trials do not establish whether opioids affect diagnostic accuracy across acute abdominal presentations. Some studies identified changes in tenderness, rigidity, or localization after analgesia, but these were not consistently associated with diagnostic error or adverse management outcomes. Adverse effects were generally short-term and included nausea, vomiting, dizziness, and sedation, although most trials were not large enough or followed long enough to reliably evaluate uncommon serious adverse events. Overall, the evidence supports an individualized approach to analgesia. Opioids remain effective when clinically indicated, but are not consistently superior to non-opioid or opioid-sparing alternatives, and analgesia should not be routinely delayed solely because of concern for diagnostic interference.

## Introduction and background

Acute abdominal pain is a common reason for emergency department visits, accounting for approximately 5-10% of all emergency department visits [1]. The differential diagnosis is broad, ranging from benign causes to potentially life-threatening surgical emergencies. Accurate and prompt diagnosis is important for avoiding serious complications, with timely pain control remaining an important component of patient-centered care. Opioids have conventionally been avoided or postponed for acute abdominal pain treatment due to concern that analgesia could alter physical examination findings and contribute to misdiagnosis or delayed treatment. However, a review of prospective trials evaluated the safety, adverse effects, and outcomes of opioid analgesia in patients with acute abdominal pain in the emergency department. No adverse outcomes or delays in diagnosis were attributed to the administration of analgesia. Based on these findings, the study concluded that opioid pain relief can be safely administered to patients presenting to the emergency department with acute abdominal pain provided no contraindications exist [2,3]. Current evidence suggests appropriately administered opioid analgesia does not substantially interfere with diagnostic accuracy [4,5]. Therefore, clinical practice has shifted toward a more patient-centered model of analgesia while continuing to balance efficient pain relief with diagnostic assessment [4].

Opioids such as fentanyl and morphine can provide rapid and effective analgesia for moderate-to-severe pain and remain commonly used in emergency medicine [6,7]. Historically, clinicians have been concerned that opioid sedation and alteration in pain profile could make examination and surgical decision-making more difficult [8]. Outcomes of a randomized controlled trial demonstrated no significant difference in diagnostic quality between patients receiving opioid analgesia and those not receiving opioids [9]. A meta-analysis also supported this, showing that early opioid analgesia does not affect diagnostic accuracy or surgical outcome [10].

An observational analysis found higher adjusted odds of a 30-day return visit for abdominal pain among patients discharged after receiving an opioid than among those receiving non-steroidal anti-inflammatory drugs (NSAIDs) or acetaminophen [11]. This raises additional questions regarding recurrent pain, medication selection, and outcomes following emergency department evaluation. Although opioid administration may alter individual components of the abdominal examination, including tenderness, localization, and rigidity, these changes have not consistently translated into impaired diagnostic accuracy or adverse clinical outcomes [12-14]. The application of these results is complicated by variation between trials, including differences in opioid dosage, time of administration, and patient populations. In addition, most comparative analgesic studies in the emergency department focus on short-term outcomes, adverse effects, and diagnostic or treatment decisions, leaving limited evidence regarding longer-term outcomes beyond the initial emergency department encounter. Aside from diagnostic concerns, opioid administration carries recognized adverse effects including nausea, sedation, and respiratory depression, as well as broader concerns for misuse and dependence [6,15]. Due to this, during their practice, emergency department providers must weigh the need to alleviate pain and suffering against potential harms associated with treatment [16]. As a response to this, providers are increasingly relying on non-opioid alternatives such as intravenous acetaminophen and NSAIDs, as well as other opioid-sparing strategies. These non-opioid medications, when not contraindicated, may demonstrate meaningful analgesia while reducing opioid exposure. NSAIDs are not without risks: gastrointestinal bleeding, renal impairment, and cardiovascular events, particularly in patients with preexisting conditions. Currently, non-opioid analgesics such as NSAIDs and acetaminophen are frequently considered alternatives or adjuncts to opioids for pain relief [17]. In cases where opioids are necessary, using the lowest effective dose and considering multimodal analgesic strategies may limit associated adverse effects [18].

Given the need to provide timely pain relief while conserving diagnostic accuracy, further evaluation of opioid and non-opioid analgesic strategies for acute abdominal pain is warranted. This systematic review and analysis evaluates randomized comparative trials of analgesia for acute non-traumatic abdominal pain in the emergency department with particular attention to opioid-containing medications. The primary objectives are to compare analgesic efficacy, adverse effects, rescue analgesia requirements, and effects on diagnostic accuracy when reported. In synthesizing evidence, this review aims to clarify the role of opioid therapy relative to non-opioid and opioid-sparing alternative strategies and support individualized evidence-based pain control for patients presenting with acute abdominal pain.

## Review

Methodology

Databases Used and Search Strategy

The literature search was conducted through PubMed, Cochrane Central Register of Controlled Trials (CENTRAL), and Embase to identify primary clinical studies evaluating opioid-containing analgesic therapy compared with non-opioid, opioid-sparing, or placebo analgesic strategies for acute, non-traumatic abdominal pain in the emergency department. The same conceptual search string was used across PubMed, CENTRAL, and Embase, with database-specific filters adapted to each platform. The search strategy was: (“abdominal pain” OR “acute abdomen” OR “acute abdominal pain” OR “acute pancreatitis” OR “appendicitis” OR “biliary colic”) AND (morphine OR fentanyl OR hydromorphone OR tramadol OR opioid OR papaveretum OR pethidine) AND (analges* OR pain) AND (“emergency department” OR “Emergency medicine” OR “ED” OR “ER”). The final database search was conducted on September 20, 2026. Filters for randomized controlled trials or clinical trials and English-language publications were applied. No publication date restriction was used.

Study Screening and Eligibility

Studies were included if they were peer-reviewed randomized comparative trials involving patients aged 18 years or older presenting to an emergency department with acute, non-traumatic abdominal pain. The age threshold was selected to maintain a defined adult emergency department population, as pediatric and adolescent patients may differ in analgesic dosing, diagnostic pathways, and management considerations. Eligible presentations included suspected appendicitis, biliary colic, acute pancreatitis, and undifferentiated acute abdominal pain. Eligible trials evaluating analgesic efficacy or safety were required to compare an opioid-containing treatment strategy with a non-opioid or opioid-sparing strategy. Opioid-sparing strategies included pharmacologic combination approaches using reduced opioid exposure as well as regional analgesic techniques. Trials evaluating the effect of opioid administration on diagnostic accuracy or clinical assessment were also eligible when the opioid intervention was compared with placebo. Studies were required to report at least one relevant outcome, including pain intensity or pain relief, rescue analgesia requirements, adverse effects, or diagnostic accuracy. English-language full text was required for final inclusion.

Studies were excluded if they were limited to renal or ureteric colic or isolated flank pain; evaluated chronic, cancer-related, traumatic, or postoperative pain; involved non-abdominal pain; included patients younger than 18 years without separately reported adult data; or consisted of reviews, editorials, case reports, conference abstracts without sufficient full-text data, or non-peer-reviewed publications. Studies comparing an opioid exclusively with another opioid were excluded because the review focused on comparisons between opioid-containing treatment and non-opioid or opioid-sparing strategies. Regional analgesic techniques were eligible when directly compared with an opioid-containing treatment strategy. Placebo-controlled opioid trials were eligible when evaluating diagnostic accuracy or the effect of analgesia on clinical assessment. Studies without relevant analgesic or diagnostic outcomes were also excluded.

For the review synthesis, analgesic efficacy and adverse effects were the main outcomes of interest. Safety outcomes were extracted as reported by each trial. We distinguished commonly reported symptoms, including nausea, vomiting, dizziness, and sedation, from serious adverse events or events requiring intervention, including clinically significant respiratory depression and hypotension. For each reported event, we recorded the number of affected patients and the treatment-group denominator when available. Because the trials used different definitions and short observation periods, absence of a reported event was not interpreted as evidence that the event could not occur. Rescue analgesia requirements and diagnostic accuracy were also evaluated when reported. When studies reported outcomes at multiple time points, the prespecified primary assessment time was prioritized, with additional clinically relevant time points reported when they contributed to interpretation of the findings.

Studies retrieved from PubMed, CENTRAL, and Embase were imported into Zotero for reference management and duplicate identification. A single reviewer performed title and abstract screening, full-text eligibility assessment, and data extraction using the inclusion and exclusion criteria. A review protocol was not prospectively registered. Preferred Reporting Items for Systematic Reviews and Meta-Analyses (PRISMA)-style study selection and flow reporting were used to improve transparency. Because of substantial clinical and methodological heterogeneity among the included trials, the evidence was synthesized narratively, and a quantitative meta-analysis was not performed.

A total of 316 records were identified across PubMed, CENTRAL, and Embase, including 43 from PubMed, 138 from CENTRAL, and 135 from Embase. Following removal of 45 duplicates, 271 unique records remained for title and abstract screening. Of these, 245 records were excluded during title and abstract screening, and 26 reports underwent full-text assessment. All 26 full-text articles were retrieved. Ten articles were excluded following full-text assessment, leaving 16 studies meeting the database search eligibility criteria. The study selection process is summarized in Figure 1.

**Figure 1:**
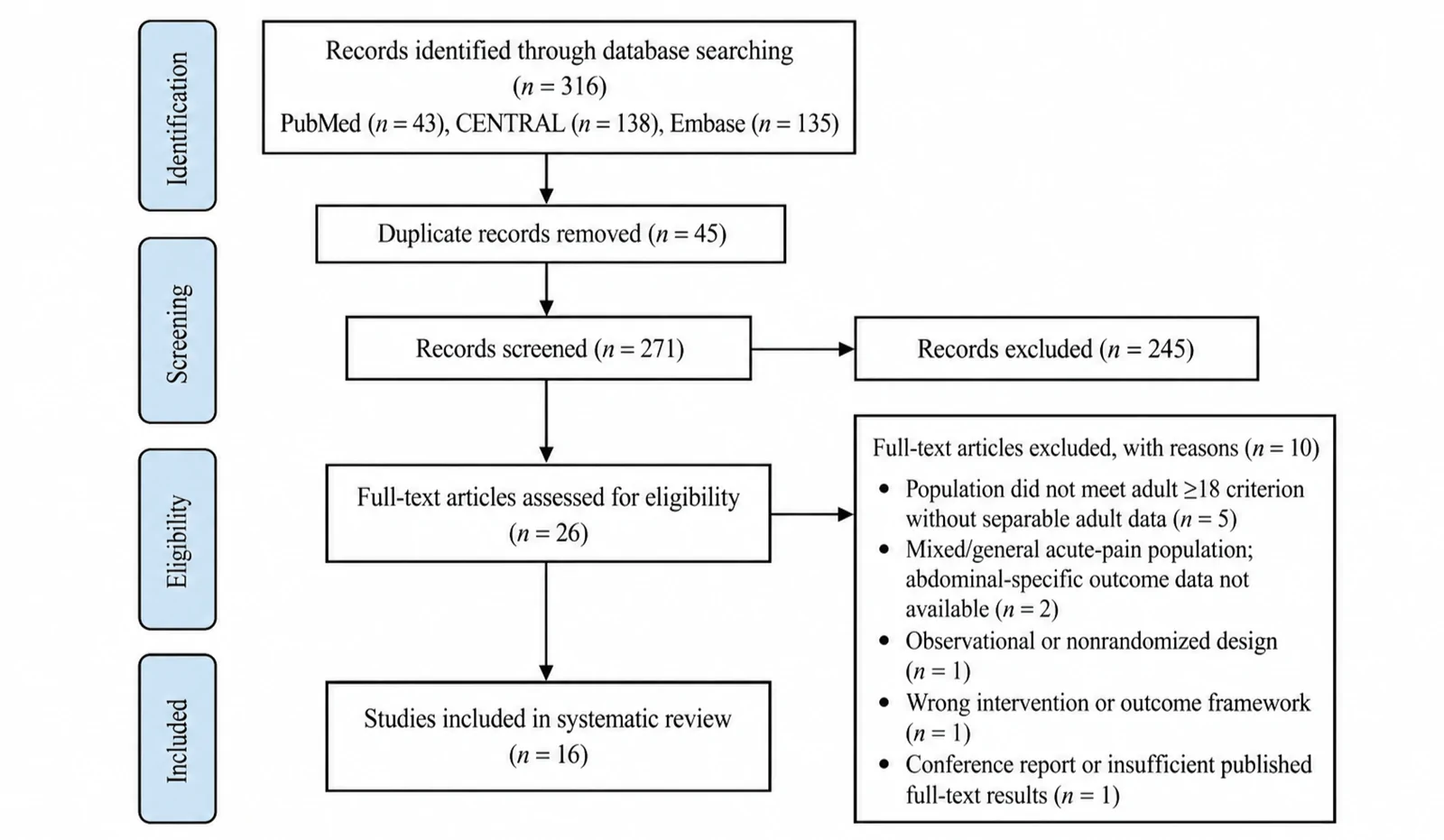
Flow diagram of the study selection process. CENTRAL: Cochrane Central Register of Controlled Trials

Risk of Bias Assessment

Risk of bias of the 16 included randomized controlled trials was assessed using the Cochrane Risk of Bias 2 (RoB 2) tool. Each of the trials was evaluated across five domains: bias arising from the randomization process, bias due to deviations from intended interventions, bias due to missing outcome data, bias in measurement of the outcome, and bias in selection of the reported result. This assessment was completed by one reviewer on the primary outcome relevant to this review: Pace and Burke [19], LoVecchio et al. [12], Dula et al. [20], Henderson et al. [21], Thomas et al. [9], Gallagher et al. [22], Olsen et al. [23], Noble et al. [24], Güngör et al. [13], Oğuztürk et al. [25], Agodirin et al. [14], Gülen et al. [26], Farnia et al. [27], Chinn et al. [28], Akhgar et al. [29] and Priyanka et al. [30] Risk-of-bias judgments were based on the RoB 2 signaling questions for the specific outcome and assessment time point evaluated in this review for the effect of assignment to intervention. The outcome, assessment time point, domain-level judgments, and supporting rationale for each trial are reported in Appendix 1. Domain-level and overall risk-of-bias judgments were summarized as low risk of bias, some concerns, or high risk of bias in Figure 2.

**Figure 2:**
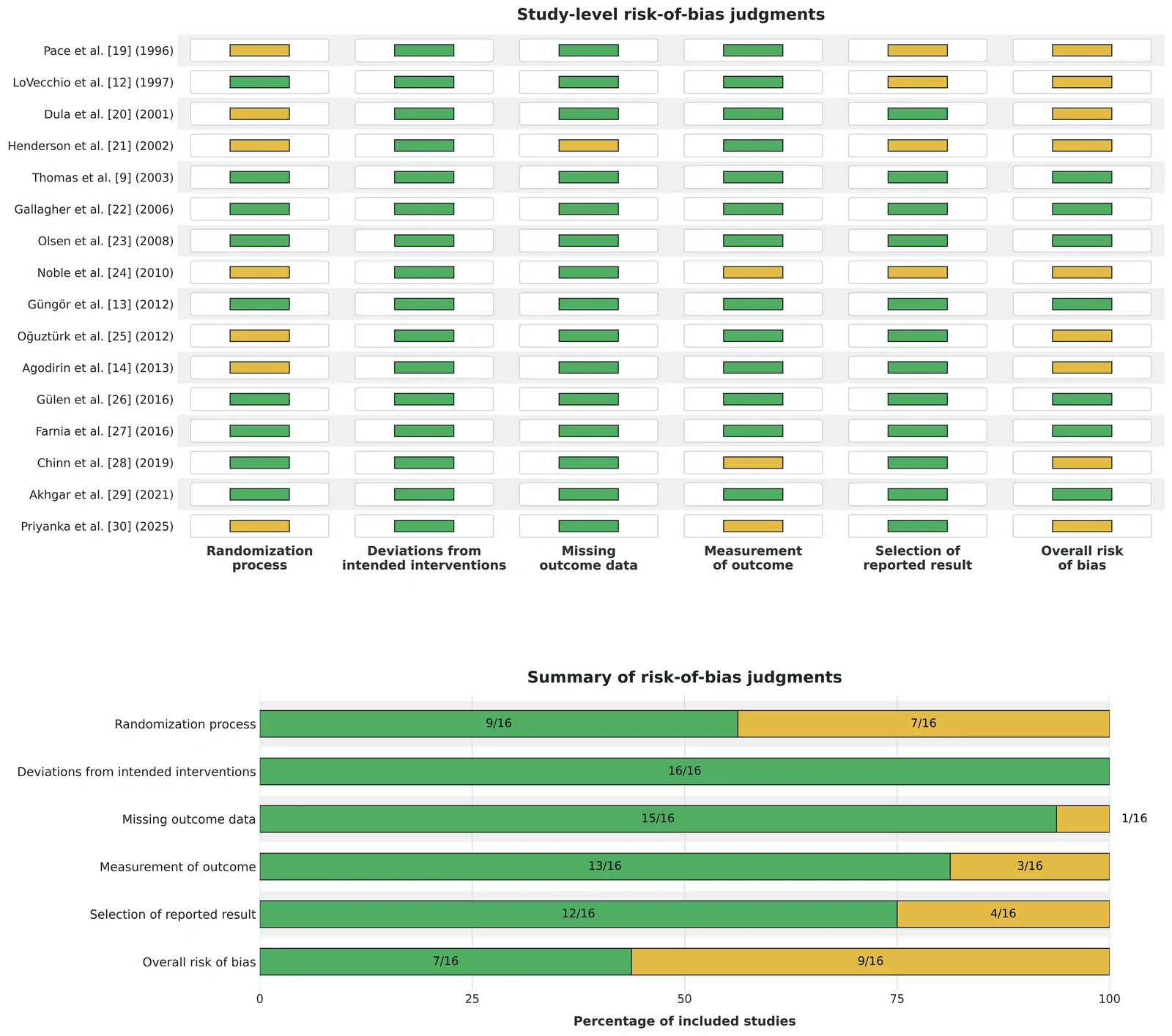
Cochrane Risk of Bias 2 assessment traffic-light and summary plot of included randomized controlled trials. Green indicates low risk of bias; Yellow indicates some concerns; Red indicates high risk of bias. The upper panel shows the risk-of-bias judgment for each study and domain, and the lower panel shows the percentage of studies in each category for each domain. The domains assessed were the randomization process, deviations from intended interventions, missing outcome data, measurement of the outcome, selection of the reported result, and overall risk of bias. No study was rated as having an overall high risk of bias.

Data Analysis

The 16 studies meeting the final criteria were synthesized qualitatively and descriptively. Comparisons were organized according to analgesic efficacy, rescue analgesia requirements, adverse effects, and diagnostic accuracy. Studies were further grouped according to clinically meaningful comparisons, including opioid versus non-opioid treatment, opioid-sparing strategies, and opioid versus placebo for studies evaluating diagnostic accuracy, and were interpreted according to the underlying clinical presentation and pain mechanism where appropriate. The direction and magnitude of treatment effects were compared using between-group effect estimates and 95% confidence intervals (CIs) when reported, rather than statistical significance alone. Risk-of-bias judgments and differences in study population, clinical presentation, intervention, comparator, and outcome assessment were considered when interpreting the consistency and applicability of the findings. A quantitative meta-analysis was not performed because of substantial clinical and methodological heterogeneity among the included trials, including differences in patient populations, underlying diagnoses, interventions, comparators, outcome measures, and assessment time points and scales. Formal certainty-of-evidence grading was not performed and is acknowledged as a limitation of this systematic review.

Results

Identified Studies

The following 16 randomized controlled trials were included and analyzed in the results after applying the inclusion and exclusion criteria: Pace and Burke [19], LoVecchio et al. [12], Dula et al. [20], Henderson et al. [21], Thomas et al. [9], Gallagher et al. [22], Olsen et al. [23], Noble et al. [24], Güngör et al. [13], Oğuztürk et al. [25], Agodirin et al. [14], Gülen et al. [26], Farnia et al. [27], Chinn et al. [28], Akhgar et al. [29] and Priyanka et al. [30] Table 1 summarizes the results of the included studies. Additional details regarding study design, population, intervention/comparator, primary endpoint, main finding, and adverse events are reported in Appendix 2.

**Table 1:** Summary of included randomized trials evaluating analgesic strategies relevant to acute abdominal pain. Scale ranges and units are reported as specified in the individual studies. CI = confidence interval; ED = emergency department; ESP = erector spinae plane; IM = intramuscular; IV = intravenous; NRS = Numeric Rating Scale; RCT = randomized controlled trial; RR = risk ratio; RUQ = right upper quadrant; SMS = sonographic Murphy sign; VAS = Visual Analog Scale

Study	Design/Population	Intervention/Comparator	Key results	Diagnostic outcome
Pace and Burke [19] (1996)	Prospective, double-blind, placebo-controlled RCT; 75 adults with acute atraumatic abdominal pain enrolled; 71 completed the study	Titrated IV morphine sulfate (up to 20 mg) vs. normal saline placebo	Mean VAS pain improvement was 3.9 ± 2.8 cm with morphine vs. 0.8 ± 1.5 cm with placebo (p < 0.01)	No significant difference in diagnostic accuracy or management errors between groups
LoVecchio et al. [12] (1997)	Prospective, randomized, placebo-controlled trial; 49 adults with acute abdominal pain randomized; 48 completed	Morphine 5 mg IV vs. morphine 10 mg IV vs. saline placebo	Significant changes in physical examination occurred in both morphine groups, but not placebo; no adverse events were attributed to analgesia	No delay in diagnosis was attributed to morphine; formal diagnostic accuracy was not the primary endpoint
Dula et al. [20] (2001)	Prospective, randomized, double-blind trial; 30 adults with confirmed acute biliary colic	Ketorolac 60 mg IM vs. meperidine 1.5 mg/kg IM (maximum 100 mg)	Mean pain relief at 30 minutes on a 1-10 VAS scale was 3.8 with ketorolac vs. 3.9 with meperidine (p = 0.962). Rescue medication was required in 12.5% vs. 28.6%, respectively	Not assessed
Henderson et al. [21] (2002)	Prospective, randomized, double-blind trial of adults aged 18–65 with biliary colic; 534 enrolled and 324 completed the protocol	Ketorolac 30 mg IV vs. meperidine 50 mg IV	No significant difference in pain control. Mean 2-hour VAS improvement was 6.2 ± 3.6 cm with ketorolac vs. 6.7 ± 3.6 cm with meperidine (p = 0.25). Meperidine caused more nausea and dizziness	Not assessed
Thomas et al. [9] (2003)	Prospective, double-blind RCT; 74 adults with undifferentiated abdominal pain (38 morphine, 36 placebo)	Titrated IV morphine sulfate vs. saline placebo	No significant differences in changes in tenderness, pain location, or overall physical examination findings between groups	Diagnostic accuracy was 64.2% with morphine vs. 66.7% with placebo (RR = 0.96, 95% CI = 0.73–1.27); no evidence of masking important examination findings
Gallagher et al. [22] (2006)	Double-blind RCT; 160 adults with acute abdominal pain randomized; 153 available for analysis	Morphine 0.1 mg/kg IV vs. placebo	Median VAS reduction at 15 minutes was 33 mm with morphine vs. 2 mm with placebo	Clinically important diagnostic accuracy was 86% vs. 85%; difference 1% (95% CI = −11% to 12%)
Olsen et al. [23] (2008)	Double-blind RCT; 46 adults with suspected biliary colic	Ketorolac 30 mg IV vs. butorphanol 1 mg IV	At 30 minutes, mean pain decreased from 7.4 to 3.1 with ketorolac and from 7.1 to 2.1 with butorphanol. Rescue analgesia was required in 6/21 (29%) ketorolac patients and 5/25 (20%) butorphanol patients (p = 0.50)	Not assessed
Noble et al. [24] (2010)	Prospective, randomized placebo-controlled study; 30 ED patients with abdominal pain undergoing RUQ ultrasound	Meperidine vs. placebo before repeat emergency physician and radiology ultrasound	Only 1/30 patients had a change in the emergency physician-assessed sonographic Murphy sign after medication	Postmedication emergency physician SMS sensitivity was 0.82 and specificity 0.95; radiology SMS sensitivity was 0.17 and specificity 1.00
Güngör et al. [13] (2012)	Prospective, randomized, double-blind, placebo-controlled trial; 80 adults ≥65 years with undifferentiated acute abdominal pain	Morphine 0.1 mg/kg IV vs. saline placebo	Mean VAS reduction was 31.6 ± 29.7 mm with morphine vs. 18.8 ± 28.6 mm with placebo. Abdominal rigidity changed more frequently after morphine	Diagnostic accuracy was 80% with morphine vs. 78% with placebo (p = 0.9802)
Oğuztürk et al. [25] (2012)	Prospective, randomized, double-blind, placebo-controlled trial; 210 adults with nontraumatic acute abdominal pain <72 hours	Tramadol 1 mg/kg IV vs. paracetamol 15 mg/kg IV vs. placebo	At 20 minutes, pain decreased by approximately 55% with tramadol and 45% with paracetamol vs. 1% with placebo; at 40 minutes, reductions were 67%, 60%, and 0%, respectively (p < 0.001)	Diagnostic accuracy was 96% with tramadol, 94% with paracetamol, and 94% with placebo; abdominal findings did not significantly differ
Agodirin et al. [14] (2013)	Prospective, randomized, double-blind, placebo-controlled trial; 95 adults aged 18–60 with nontraumatic acute abdomen	Tramadol 100 mg IV vs. water-for-injection placebo	Pain improvement was significantly greater with tramadol (p = 0.001), with improved abdominal palpation findings (p = 0.02)	Changes in diagnosis (p = 0.01) and treatment decisions (p = 0.03) were more frequent after tramadol, but most changes were beneficial; diagnostic accuracy was not adversely affected
Gülen et al. [26] (2016)	Single-blind, randomized controlled trial; 90 adults with acute pancreatitis	Paracetamol 1 g IV vs. dexketoprofen 50 mg IV vs. tramadol 1 mg/kg IV	Median VAS reductions at 30 minutes were approximately 41.5, 40.5, and 45.5 points, respectively; no significant between-group difference (p = 0.38)	Not assessed
Farnia et al. [27] (2016)	Double-blind RCT; 87 adults with confirmed biliary colic	Morphine 0.05 mg/kg IV + paracetamol 1 g IV vs. morphine 0.1 mg/kg IV + saline placebo	Mean VAS on a 0–10 scale was 2.16 vs. 2.51 at 15 minutes (p = 0.38) and 1.66 vs. 2.14 at 30 minutes (p = 0.19). Two combination-group patients and no standard-dose morphine patients required rescue fentanyl	Not assessed
Chinn et al. [28] (2019)	Double-blind RCT; 154 adults with acute severe abdominal pain	Lidocaine 120 mg IV vs. hydromorphone 1 mg IV; second assigned dose permitted if requested	At 90 minutes, mean pain improvement was 3.8 with lidocaine vs. 5.0 with hydromorphone; difference 1.2 points (95% CI = 0.3–2.2). Rescue analgesia was required in 51% vs. 26%	Not assessed
Akhgar et al. [29] (2021)	Double-blind RCT; 104 adults aged 18–64 with RUQ pain suspected to represent biliary colic	Lidocaine 100 mg IV vs. morphine 5 mg IV	Lidocaine produced faster early pain reduction, while morphine had lower pain scores at 30 minutes. There was no significant difference at the prespecified 60-minute primary endpoint	Not assessed
Priyanka et al. [30] (2025)	Single-blind RCT of adults aged 18–70 with acute pancreatitis and NRS >4/10	Bilateral ESP block with ropivacaine 0.375% (40 mL) vs. tramadol 1 mg/kg IV every 6 hours; fentanyl rescue in both groups	ESP block produced significantly lower pain scores through 16 hours and markedly reduced rescue fentanyl use (14% vs. 94%, p < 0.001)	Not assessed

Opioid Versus Non-opioid Analgesia

Several trials compared opioid analgesics directly with non-opioid medications, with results varying according to the medication, underlying condition, and time of assessment.

In biliary colic, Dula et al. [20] compared intramuscular meperidine with ketorolac and found nearly identical mean pain reductions at 30 minutes, 3.9 and 3.8 points, respectively (p = 0.962). Rescue analgesia was required in 28.6% of patients receiving meperidine and 12.5% receiving ketorolac, although the difference was not statistically significant. Henderson et al. [21] similarly compared intravenous meperidine with intravenous ketorolac in biliary colic. Mean Visual Analog Scale (VAS) improvement at two hours was 6.7 cm with meperidine and 6.2 cm with ketorolac (p = 0.25), without a significant difference in analgesic effect.

Olsen et al. [23] compared butorphanol with ketorolac in patients with suspected biliary colic. Butorphanol produced more rapid pain reduction at 15 minutes, while pain scores at 30 minutes were not significantly different between groups. Rescue analgesia was required in 20% of patients receiving butorphanol and 29% receiving ketorolac (p = 0.50).

In acute pancreatitis, Gülen et al. [26] compared tramadol with paracetamol and dexketoprofen. Median VAS reductions at 30 minutes were 45.5, 41.5, and 40.5 points, respectively, with no statistically significant between-group difference. These findings did not demonstrate an analgesic advantage for tramadol over either non-opioid comparator within the 30-minute assessment period.

Two trials compared opioids with intravenous lidocaine. Chinn et al. [28] demonstrated greater pain reduction with hydromorphone than lidocaine at 90 minutes. Mean improvement was 5.0 points with hydromorphone compared with 3.8 with lidocaine, for a between-group difference of 1.2 points (95% CI = 0.3-2.2). In addition, 61% of hydromorphone patients compared with 39% of lidocaine patients achieved at least a 50% improvement in pain, a difference of 22% (95% CI = 7%-37%). In contrast, Akhgar et al. [29] found no significant difference between morphine and lidocaine at the prespecified 60-minute primary endpoint in suspected biliary colic. Differences were observed at earlier time points, with lower Numerical Rating Scale (NRS) scores after lidocaine at 10 minutes and after morphine at 30 minutes.

Oğuztürk et al. [25] compared tramadol, paracetamol, and placebo in undifferentiated acute abdominal pain. Both active medications produced substantial reductions in VAS pain scores compared with placebo at 20 and 40 minutes, with a larger reduction observed in the tramadol group. These findings demonstrate effective short-term analgesia with both opioid and non-opioid treatment rather than a consistent advantage of opioid therapy across all comparative trials.

Opioid Versus Regional Block

Priyanka et al. [30] compared intravenous tramadol with bilateral erector spinae plane block using ropivacaine in patients with acute pancreatitis. The erector spinae plane block produced lower NRS pain scores for 16 hours. At one hour, the mean NRS was 1.54 ± 0.67 with the erector spinae plane block compared with 3.18 ± 0.66 with tramadol (p < 0.001). The difference was maintained at 4, 8, 12, and 16 hours but was no longer statistically significant at 24 hours. Rescue fentanyl was required in 14% of patients receiving the erector spinae plane block compared with 94% receiving tramadol (p < 0.001). Mean rescue fentanyl consumption among those requiring it was also lower with the erector spinae plane block, 66.14 ± 4.63 μg compared with 113.17 ± 33.24 μg. This trial demonstrated an opioid-sparing effect from regional analgesia in acute pancreatitis; however, it represents a different analgesic approach from the systemic medication comparisons and should be interpreted separately.

Opioid Versus Opioid-Sparing Treatment

Farnia et al. [27] evaluated whether adding intravenous paracetamol to a reduced dose of morphine could maintain analgesia while decreasing opioid exposure in patients with biliary colic. Patients receiving morphine 0.05 mg/kg with paracetamol 1 g had a mean VAS of 2.16 at 15 minutes compared with 2.51 with morphine 0.1 mg/kg alone. The mean difference was -0.35 points (95% CI = -1.15 to 0.45; p = 0.38). At 30 minutes, mean VAS scores were 1.66 and 2.14, respectively, with a mean difference of -0.48 points (95% CI = -1.20 to 0.24; p = 0.19). Two patients in the opioid-sparing group and none in the standard-dose morphine group required rescue fentanyl.

The trial did not identify a statistically significant between-group difference in pain scores, although the CIs do not establish equivalence between the strategies. The findings provide evidence that lower-dose morphine combined with paracetamol can produce substantial short-term pain reduction, while the study was not designed to establish formal equivalence or non-inferiority.

Opioid Versus Placebo

The placebo-controlled trials consistently demonstrated that opioids produced short-term analgesia, while the magnitude of benefit varied between studies.

Pace and Burke [19] demonstrated significantly greater pain reduction after titrated intravenous morphine compared with saline placebo. LoVecchio et al. [12] similarly found significant reductions in patient-reported pain following both 5 mg and 10 mg intravenous morphine, while placebo produced substantially less change. Gallagher et al. [22] reported a median VAS reduction of 33 mm at 15 minutes with morphine compared with 2 mm with placebo.

In elderly patients with undifferentiated abdominal pain, Güngör et al. [13] reported a mean VAS reduction of 31.6 ± 29.7 mm after morphine compared with 18.8 ± 28.6 mm after placebo. Agodirin et al. [14] also demonstrated greater improvement in pain after intravenous tramadol than placebo (p = 0.001). Oğuztürk et al. [25] demonstrated substantial pain reduction with tramadol compared with placebo at both 20 and 40 minutes.

Thomas et al. [9] and Noble et al. [24] primarily evaluated the effect of opioid administration on diagnostic assessment rather than comparative analgesic efficacy. Their findings are therefore considered more directly in the diagnostic accuracy section.

Analgesic Efficacy and Rescue Analgesia

Across the included studies, opioid medications consistently produced reductions in acute pain from baseline; however, greater efficacy than an active non-opioid comparator was not demonstrated in every clinical presentation. Hydromorphone demonstrated greater pain reduction than intravenous lidocaine in undifferentiated abdominal pain, while meperidine and ketorolac produced similar reductions in biliary colic, and tramadol did not demonstrate a significant advantage over paracetamol or dexketoprofen in acute pancreatitis.

Rescue analgesic requirements also varied. Chinn et al. [28] reported rescue analgesia in 51% of patients receiving lidocaine compared with 26% receiving hydromorphone, a difference of 25% (95% CI = 10%-40%). In the Akhgar [29] trial, rescue medication was more frequently required in the lidocaine group than the morphine group, although the difference was not statistically significant. In contrast, Dula et al. [20] reported rescue medication in 28.6% of meperidine patients compared with 12.5% of ketorolac patients. Olsen et al. [23] reported rescue analgesia in 20% of patients receiving butorphanol and 29% receiving ketorolac.

The largest difference in rescue analgesic use was reported with regional analgesia. Priyanka et al. [30] found rescue fentanyl was required in 94% of patients treated with tramadol compared with 14% following erector spinae plane block. Farnia et al. [27] reported rescue fentanyl in two patients receiving low-dose morphine with paracetamol and none receiving standard-dose morphine. In the placebo-controlled elderly population studied by Güngör et al. [13], rescue treatment was required in 46% of morphine patients and 54% of placebo patients, a difference of 8% (95% CI = -15% to 30%).

Overall, these findings demonstrate variability in both pain reduction and the need for additional analgesia. The benefit of an opioid-containing strategy was dependent on the clinical condition and comparator rather than representing a consistent advantage across the included trials.

Adverse Events

Adverse event definitions and reporting varied across trials, and observation periods were generally short. Reported symptoms most often included nausea, vomiting, dizziness, and sedation. Serious events, including respiratory depression and clinically significant hypotension, were assessed or reported inconsistently. The absence of such events in individual trials cannot establish their frequency or demonstrate equivalent safety between treatments.

In the larger biliary colic trial by Henderson et al. [21], nausea occurred in 16.4% of patients receiving meperidine compared with 6.8% receiving ketorolac (p = 0.009), while dizziness occurred in 17.9% and 6.8%, respectively (p = 0.003). Drowsiness occurred in 42.1% of meperidine patients and 34.6% of ketorolac patients. Olsen et al. [23] also identified differences in adverse effect profiles: butorphanol produced more sedation than ketorolac (36% vs. 5%) and more dizziness (28% vs. 0%), while nausea was more frequent with ketorolac (24% vs. 4%).

Chinn et al. [28] reported at least one patient-reported symptom in 36% of hydromorphone patients and 30% of lidocaine patients, a difference of 6% (95% CI = -8% to 21%). Dizziness was more frequent with hydromorphone (18% vs. 5%), while there were no serious adverse events and no patient required naloxone.

The opioid-sparing trial by Farnia et al. [27] reported low frequencies of nausea, vomiting, sedation, and dizziness in both groups. No hypotension, allergic reactions, or respiratory depression occurred in either the low-dose morphine plus paracetamol group or the standard-dose morphine group.

Among placebo-controlled studies, Güngör et al. [13] reported an adverse event rate of 28% with morphine and 12% with placebo, a difference of 16% (95% CI = -4% to 36%; p = 0.10). Nausea or vomiting occurred in 12.8% and 4.9%, respectively, while hypotension occurred in one morphine patient and no placebo patients. Oğuztürk et al. [25] found no significant difference in adverse events between tramadol, paracetamol, and placebo (p = 0.80), and no patient required intervention for a medication-related adverse effect. Thomas et al. [9] reported no study medication side effects, and Gallagher et al. [22] reported that no patient required naloxone.

Akhgar et al. [29] reported dizziness in five patients receiving lidocaine and vomiting in four receiving morphine, with no event requiring physician intervention. Priyanka et al. [30] reported no complications or adverse drug reactions in either the erector spinae plane block or tramadol groups.

The available trials therefore identify primarily minor and self-limited adverse effects. However, the relatively small sample sizes, short observation periods, and inconsistent reporting of outcomes such as respiratory depression, hypotension, and emergency department length of stay limit conclusions regarding uncommon or longer-term harms.

Diagnostic Accuracy

Diagnostic outcomes were evaluated in several of the earlier placebo-controlled studies and generally did not demonstrate a reduction in diagnostic accuracy following opioid administration, although the precision and method of assessment differed between trials.

Pace and Burke [19] found no statistically significant difference in diagnostic measures between morphine and placebo. The emergency physician’s primary diagnosis agreed with the final diagnosis in 80% of morphine patients and 61% of placebo patients, while three major diagnostic errors occurred in each group. Morphine did not result in loss of peritoneal signs.

Thomas et al. [9] found diagnostic accuracy of 64.2% with morphine and 66.7% with placebo (risk ratio = 0.96, 95% CI = 0.73-1.27). There was also no evidence that morphine masked clinically important physical examination findings.

Gallagher et al. [22] reported clinically important diagnostic accuracy of 86% with morphine and 85% with placebo, corresponding to a difference of 1% (95% CI = -11% to 12%). The CI was compatible with differences in either direction and therefore does not establish equivalence, but the observed results did not demonstrate impaired diagnostic accuracy following morphine administration.

In elderly patients, Güngör et al. [13] reported diagnostic accuracy of 80% with morphine and 78% with placebo, a difference of 2% (95% CI = -7% to 13%; p = 0.9802). Morphine was associated with a greater change in abdominal rigidity, demonstrating that individual physical examination findings may change despite the absence of a demonstrated difference in overall diagnostic accuracy.

Oğuztürk et al. [25] reported diagnostic accuracy of 96% with tramadol, 94% with paracetamol, and 94% with placebo. Final diagnosis changed in 11 of 210 patients, while abdominal examination findings did not significantly differ between treatment groups after analgesic administration.

Agodirin et al. [14] found that changes in provisional diagnosis occurred more frequently after tramadol than placebo (10/46 vs. 2/49; p = 0.01). Eight of the changes following tramadol moved the diagnosis toward the final diagnosis, while two produced diagnostic confusion. Changes in treatment decisions were also more frequent after tramadol, but most were toward the definitive treatment. These findings demonstrate that analgesia may alter clinical assessment without necessarily producing a net reduction in diagnostic accuracy.

LoVecchio et al. [12] also demonstrated that morphine could alter tenderness and localization on physical examination. However, no adverse events or delays in diagnosis were attributed to opioid administration.

Noble et al. [24] specifically evaluated the sonographic Murphy sign. Only one of 30 emergency physician assessments changed following study medication. The post-medication emergency physician-performed sonographic Murphy sign had a sensitivity of 0.82 and a specificity of 0.95 for cholecystitis. In contrast, nine radiology examinations had a non-diagnostic sonographic Murphy sign, including three in which this was explicitly attributed to administration of pain medication. These findings suggest that the effect of analgesia on diagnostic assessment may depend partly on the examination method and operator.

Taken together, the diagnostic studies did not demonstrate a consistent reduction in diagnostic accuracy with opioid administration. However, several studies demonstrated changes in individual physical examination findings, and CIs around diagnostic estimates were often wide. The available evidence therefore supports that analgesia can be administered during evaluation of acute abdominal pain without a demonstrated increase in diagnostic error in the included trials, while avoiding the stronger conclusion that opioids have been proven to have no effect on diagnostic assessment.

Overall, the included studies demonstrated that opioids are effective analgesics for acute abdominal pain but are not consistently superior to non-opioid, opioid-sparing, or regional analgesic strategies. The observed differences between studies appeared to depend on the clinical presentation, comparator medication, and timing of outcome assessment. These findings support an individualized approach to analgesia that considers expected analgesic benefit, adverse effects, contraindications, and the underlying cause of abdominal pain.

Discussion

Principal Findings

The purpose of this systematic review was to evaluate the efficacy and safety of opioid analgesia for acute abdominal pain while comparing opioid-containing treatment strategies with non-opioid, opioid-sparing, and regional analgesic alternatives. Diagnostic accuracy and the effect of analgesia on clinical assessment were also considered. Across the 16 included studies, opioids consistently provided short-term analgesia; however, they did not demonstrate a consistent advantage over alternative analgesic strategies. The magnitude of benefit varied according to the medication, underlying clinical condition, comparator, and time at which the outcome was assessed.

The included trials support an important role for opioids in the treatment of moderate-to-severe acute abdominal pain, particularly when rapid analgesia is required. Morphine demonstrated greater pain reduction than placebo in several studies, and hydromorphone produced greater pain improvement than intravenous lidocaine in the trial by Chinn et al. [28]. However, other direct comparisons demonstrated less separation between opioid and non-opioid treatment. Ketorolac provided pain reduction similar to meperidine in biliary colic; tramadol did not demonstrate a clear analgesic advantage over paracetamol or dexketoprofen in acute pancreatitis; and lidocaine did not significantly differ from morphine at the primary 60-minute endpoint in the Akhgar et al. [29] trial. The opioid-sparing strategy evaluated by Farnia et al. [27] also produced substantial pain reduction using lower-dose morphine combined with paracetamol, although the study was not designed to demonstrate formal equivalence to standard-dose morphine.

Regional analgesia provided another strategy for reducing opioid requirements. Priyanka et al. [30] demonstrated lower pain scores and substantially lower rescue fentanyl requirements following an erector spinae plane block compared with intravenous tramadol in acute pancreatitis. This finding is limited to a single condition-specific trial but demonstrates that effective analgesia for acute abdominal pain does not necessarily require increasing systemic opioid exposure.

The additional trials provide a broader basis for assessing whether opioid analgesia interferes with diagnosis. They did not show a consistent increase in diagnostic error, although some reported changes in tenderness, rigidity, or localization after analgesia. The estimates remain imprecise: for example, Gallagher et al. [22] reported diagnostic accuracy of 86% with morphine and 85% with placebo, a difference of 1 percentage point (95% CI = -11 to 12). These findings should also be interpreted in the context of contemporary emergency practice, where greater availability of imaging, particularly CT and point-of-care ultrasonography, provides additional diagnostic information and may reduce reliance on subtle changes in physical examination findings alone. However, imaging does not replace clinical assessment and reassessment. Because the trials enrolled different abdominal presentations and assessed diagnostic outcomes differently, they cannot establish that opioids have no clinically important effect on diagnostic accuracy across all presentations.

Risk-of-bias assessment found seven studies to be at low overall risk and nine to have some concerns, with no study assessed as having an overall high risk of bias. These findings, combined with differences in patient populations, interventions, and outcome assessment, require that the results be interpreted as a heterogeneous body of evidence rather than as support for a single analgesic strategy.

Analgesic Strategy and Safety

The variability between trials suggests that the selection of analgesia for acute abdominal pain should be guided by the clinical presentation rather than by the assumption that one medication class is consistently superior. This is particularly evident in biliary colic, where several different treatment approaches were studied. Dula et al. [20] and Henderson et al. [21] found little difference in pain reduction between ketorolac and meperidine, while Olsen et al. [23] found more rapid early pain relief with butorphanol but similar pain scores to ketorolac by 30 minutes. Akhgar et al. [29] similarly found differences between lidocaine and morphine at earlier assessment times without a significant difference at the prespecified 60-minute primary endpoint. Together, these studies demonstrate that both the condition being treated and timing of assessment can substantially influence apparent comparative effectiveness.

A similar pattern was observed in acute pancreatitis. Gülen et al. [26] found substantial pain reduction with tramadol, paracetamol, and dexketoprofen without a statistically significant difference between groups at 30 minutes. Priyanka et al. [30], however, demonstrated substantially lower pain scores and rescue fentanyl requirements using an erector spinae plane block compared with repeated intravenous tramadol. These findings should be interpreted in relation to the underlying pain mechanism. Acute pancreatitis and biliary colic have substantial visceral pain components, whereas irritation of the parietal peritoneum produces more localized somatic pain. The relative effectiveness of systemic opioids and regional analgesic techniques may therefore differ across clinical presentations. These studies address different analgesic approaches and should not be considered equivalent comparisons, and findings from the condition-specific erector spinae plane block trial should not be generalized to all causes of acute abdominal pain, but they demonstrate the potential role of non-opioid and regional strategies in selected patients.

Rescue analgesia provides additional information beyond initial pain score reduction. Chinn et al. [28] found that patients receiving intravenous lidocaine required rescue analgesia more frequently than those receiving hydromorphone, supporting a greater sustained analgesic effect from hydromorphone in that trial. Conversely, regional analgesia in the Priyanka et al. [30] trial substantially reduced rescue fentanyl requirements compared with tramadol. Other trials, including Dula et al. [20], Olsen et al. [23], Farnia et al. [27], Akhgar et al. [29], and Güngör et al. [13], demonstrated smaller or statistically uncertain differences in rescue medication use. The overall findings again suggest that opioid reduction alone should not be considered evidence of improved analgesia unless adequate pain control is maintained.

Safety outcomes also varied according to the analgesic used. Most reported adverse effects were minor and self-limited, including nausea, vomiting, dizziness, and sedation. Henderson et al. [21] reported higher rates of nausea and dizziness with meperidine than ketorolac. Olsen et al. [23] found greater sedation and dizziness with butorphanol, while nausea was more frequently reported with ketorolac. Chinn et al. [28] found similar overall rates of medication-associated symptoms with lidocaine and hydromorphone, although dizziness was more common with hydromorphone. No serious adverse events or naloxone administration were reported in that trial.

Farnia et al. [27] reported low rates of nausea, vomiting, dizziness, and sedation with both opioid-containing regimens and no hypotension, allergic reactions, or respiratory depression. Güngör et al. [13] observed a higher overall proportion of adverse events with morphine than placebo, although the CI was wide and the difference was not statistically significant. Akhgar et al. [29] reported only transient adverse events that did not require physician intervention, while Priyanka et al. [30] reported no complications or adverse drug reactions in either treatment group.

The limited reporting of serious adverse events does not establish that the analgesic strategies have equivalent safety. Most trials had relatively small sample sizes and brief observation periods and were therefore inadequately powered to identify uncommon complications such as clinically significant respiratory depression or hypotension. Emergency department length of stay and other measures of throughput were also inconsistently reported. Safety should therefore be considered alongside expected analgesic benefit, patient characteristics, contraindications, and the clinical condition being treated.

Comparison With Previous Evidence

The findings of this review are consistent with the broader literature regarding the historical concern that opioid administration may interfere with the diagnosis of acute abdominal pain. Earlier reviews by Ranji et al. [3] and subsequent analyses of early analgesia have evaluated whether opioid administration changes the physical examination or increases diagnostic and management errors. The additional randomized trials included in the present review strengthen this body of evidence by demonstrating that changes in individual examination findings do not necessarily result in a reduction in overall diagnostic accuracy.

The 2019 systematic review and network meta-analysis by Gavriilidis et al. [4] also evaluated opioid administration before definitive surgical diagnosis, reflecting the same long-standing clinical question. The findings of the current review are generally consistent with this previous work. Rather than demonstrating a pattern of increased diagnostic error following analgesia, the randomized studies demonstrate that morphine and other analgesics can be administered during the diagnostic evaluation without a consistent adverse effect on the final diagnosis. This does not mean that the physical examination is unaffected. LoVecchio et al. [12], Güngör et al. [13], and Agodirin et al. [14] demonstrated that individual findings or the clinician’s assessment may change after pain relief. These changes should be expected when pain and guarding improve and do not in themselves establish that diagnostic accuracy has been impaired.

This distinction is important when interpreting the older concern that analgesia may "mask" an acute abdomen. The more clinically relevant outcome is not whether tenderness changes after treatment but whether analgesia results in incorrect diagnosis, delayed necessary treatment, or clinically important adverse outcomes. Pace and Burke [19], Thomas et al. [9], and Gallagher et al. [22] did not demonstrate a reduction in overall diagnostic accuracy following morphine administration, while Oğuztürk et al. [25] reported similar diagnostic accuracy across tramadol, paracetamol, and placebo. Agodirin et al. [14] further demonstrated that changes in diagnosis following tramadol were more commonly changes toward the final diagnosis than toward diagnostic confusion.

More recent literature has increasingly focused on reducing unnecessary opioid exposure while maintaining adequate analgesia rather than withholding analgesia because of concern for diagnostic interference. Heard et al. [31], in a large retrospective cohort of patients with non-specific abdominal pain, found ketorolac administration to be associated with modestly lower odds of receiving intravenous opioids, although the study did not assess patient-reported analgesic efficacy. This distinction is important because reduction in opioid administration should not automatically be interpreted as equivalent pain control.

Broader acute pain literature has similarly emphasized multimodal treatment using medications such as acetaminophen and NSAIDs where clinically appropriate. Reviews by Pergolizzi et al. [17] and Hyland et al. [18] provide additional context for the increasing use of non-opioid and opioid-sparing strategies, although this evidence is not specific to acute abdominal pain and should therefore be considered indirect. The trials included in the present review provide more direct support for this approach by demonstrating that selected non-opioid, opioid-sparing, and regional strategies can provide effective analgesia in specific abdominal conditions.

Professional guidance is consistent with this approach. The American College of Emergency Physicians (ACEP) recommends prompt, safe, and patient-specific treatment of acute pain using multimodal analgesic strategies [32]. World Society of Emergency Surgery (WSES) guidance for acute pancreatitis states that there is no evidence supporting restriction of pain medication [33]. These recommendations further support providing timely analgesia while tailoring medication selection to the clinical presentation.

The current evidence therefore shifts the clinical question away from whether analgesia should be withheld before diagnosis and toward which analgesic strategy provides the most appropriate balance of pain relief, rescue medication requirements, and adverse effects for the individual presentation.

Implications for Practice

Effective analgesia should remain an important component of the early management of patients presenting with acute abdominal pain. The included evidence does not support routinely delaying opioid administration solely because of concern that analgesia will prevent an accurate diagnostic evaluation. Although some examination findings may change following pain relief, the available randomized evidence did not demonstrate a consistent increase in diagnostic error.

Opioids remain an appropriate treatment when clinically indicated, particularly in patients with moderate-to-severe pain or when alternative medications are contraindicated or insufficient. However, the evidence also does not support routine opioid superiority across all presentations. NSAIDs, acetaminophen, intravenous lidocaine, opioid-sparing combinations, and regional analgesia may provide effective alternatives depending on the underlying condition and patient characteristics.

The choice of analgesic should therefore be individualized according to the likely cause of pain, pain severity, contraindications, expected adverse effects, and response to initial treatment. Biliary colic, acute pancreatitis, and undifferentiated abdominal pain represent different clinical conditions and should not be expected to respond identically to the same analgesic strategy. Rescue medication requirements and adverse effects should also be considered rather than relying only on a single pain score at one assessment time.

Multimodal analgesia may provide a reasonable approach when it allows adequate pain control while reducing unnecessary opioid exposure. However, opioid-sparing should not become undertreatment of pain. The goal remains adequate and timely analgesia using the most appropriate treatment for the individual patient rather than avoidance of a specific medication class.

Limitations

This review has several limitations that should be considered when interpreting its findings. The included studies were clinically heterogeneous, ranging from undifferentiated acute abdominal pain to biliary colic and acute pancreatitis. These conditions have different underlying mechanisms of pain and were treated with different medications, doses, and routes of administration. Direct comparison between studies is therefore limited, and the findings should not be interpreted as demonstrating that one analgesic strategy is preferable across all acute abdominal presentations.

Several trials were conducted at a single center or enrolled relatively small study populations. This reduced the precision of individual estimates and limited the ability to identify uncommon adverse events. Safety outcomes were also inconsistently defined and reported, with most studies focused primarily on short-term nausea, vomiting, dizziness, or sedation. Serious respiratory depression, hypotension and other uncommon complications could not be reliably evaluated from the available sample sizes and follow-up periods.

Outcome assessment also differed substantially. Studies used different pain scales, medication doses, assessment times, and definitions of clinically important pain relief. Some trials evaluated outcomes within 15 to 30 minutes while others followed pain over several hours. This clinical and methodological heterogeneity, combined with differences in diagnosis, intervention, and comparator, limited the appropriateness of quantitative pooling. Within clinically relevant comparison groups, there were too few studies evaluating sufficiently similar populations, interventions, comparators, outcomes, and assessment time points to support a meaningful meta-analysis; therefore, the evidence was synthesized narratively.

Although the included evidence provides considerable information regarding diagnostic assessment, diagnostic outcomes were not measured in the same manner across studies. Some trials evaluated overall diagnostic accuracy, while others examined physical examination findings, changes in diagnosis, surgical decision-making, or the sonographic Murphy sign. These outcomes provide complementary information but cannot be considered interchangeable.

Most included studies also focused on short-term outcomes. Emergency department length of stay, return visits, patient satisfaction, functional recovery, and longer-term clinical outcomes were inconsistently reported. Observational work such as Ginsberg et al. [11] has examined later outcomes including return visits, but comparable longer-term outcomes were not consistently available from the included randomized trials.

The review was restricted to English-language studies, which may have excluded otherwise relevant evidence. A single reviewer performed screening, eligibility assessment, data extraction, and risk-of-bias assessment, increasing the potential for selection or assessment bias. The review protocol was not prospectively registered, and formal certainty-of-evidence grading was not performed. Risk-of-bias assessment identified some concerns in nine of the 16 included studies, which should also be considered when interpreting the strength of the overall findings.

## Conclusions

Acute abdominal pain is a common emergency department presentation, and this systematic review found that opioids provide effective short-term analgesia when clinically indicated but are not consistently superior to non-opioid, opioid-sparing, or regional analgesic strategies. Comparative effectiveness varied according to the underlying clinical condition, comparator therapy, and timing of assessment, supporting an individualized rather than uniform approach to analgesic selection. Across the included randomized trials, opioid administration was not associated with a consistent reduction in diagnostic accuracy, although analgesia sometimes altered individual physical examination findings; in contemporary practice, CT and point-of-care ultrasonography provide additional diagnostic information that may reduce reliance on subtle examination changes alone. Non-opioid, opioid-sparing, and regional techniques can provide effective alternatives in selected patients, while the available safety data were generally limited to short-term, self-limited adverse effects and were insufficient to exclude uncommon serious events. Overall, the evidence supports timely, multimodal, and patient-specific analgesia in which opioids remain an appropriate option when indicated, while future research should focus on comparative effectiveness, rescue analgesia, uncommon adverse events, and longer-term patient-centered outcomes.

## Appendices

**Table 2:** Appendix 1: Detailed Cochrane RoB 2 assessment. Risk of bias was assessed for the primary result from each included randomized trial that was most relevant to this review. The effect of interest was the effect of assignment to intervention. Judgments were categorized as low risk or some concerns. Brief reasons are provided where some concerns were identified. VAS = Visual Analog Scale; NRS = Numeric Rating Scale; ESP = erector spinae plane

Study	Outcome/Time point assessed	Randomization process	Deviations from intended interventions	Missing outcome data	Measurement of outcome	Selection of reported result	Overall risk of bias
Pace and Burke [19] (1996)	Emergency-physician diagnostic accuracy 15 minutes after completion of titrated study solution	Some concerns – computer-generated randomization was reported, but allocation concealment before assignment was not clearly described	Low risk	Low risk	Low risk	Some concerns – several eligible diagnostic measures were reported, including primary diagnosis, differential diagnosis, disposition, and diagnostic/management errors, without one diagnostic result clearly prioritized	Some concerns
LoVecchio et al. [12] (1997)	Change in abdominal tenderness/localization 15 minutes after study-drug infusion	Low risk	Low risk	Low risk	Low risk	Some concerns – several physical examination findings were evaluated, and no single examination result was clearly identified as the primary result	Some concerns
Dula et al. [20] (2001)	Pain relief from baseline to 30 minutes	Some concerns – a posted randomized schedule was used, but sequence generation and concealment before assignment were not described in sufficient detail	Low risk	Low risk	Low risk	Low risk	Some concerns
Henderson et al. [21] (2002)	VAS pain reduction at 2 hours	Some concerns – a computer-generated random-number list was used, but allocation concealment before assignment was not clearly described	Low risk	Some concerns – 534 patients were enrolled but only 324 completed the protocol and had completed study forms	Low risk	Some concerns – pain was assessed using both VAS and VRS at multiple time points without a single primary pain result clearly prioritized	Some concerns
Thomas et al. [9] (2003)	Diagnostic accuracy based on the 60-minute post-medication diagnostic assessment compared with final diagnosis	Low risk	Low risk	Low risk	Low risk	Low risk	Low risk
Gallagher et al. [22] (2006)	Clinically important diagnostic accuracy; provisional diagnosis at 15 minutes compared with final diagnosis obtained during ≥6-week follow-up	Low risk	Low risk	Low risk	Low risk	Low risk	Low risk
Olsen et al. [23] (2008)	Pain score at 30 minutes; 15- and 30-minute pain assessments were prespecified primary outcomes	Low risk	Low risk	Low risk	Low risk	Low risk – the prespecified 15- and 30-minute pain outcomes were both reported	Low risk
Noble et al. [24] (2010)	Emergency physician assessed sonographic Murphy sign after study medication; first repeat assessment 10 minutes after medication	Some concerns – patients were randomized to meperidine or placebo, although the method of sequence generation and allocation concealment was not described	Low risk	Low risk – all 30 patients contributed to the emergency-physician post-medication sonographic Murphy sign assessment	Some concerns – the authors reported that blinding of the emergency physician sonographer was imperfect because drug effects could reveal treatment assignment	Some concerns – several eligible sonographic assessments were performed, including repeat emergency physician and radiology assessments, without a single analysis clearly prioritized	Some concerns
Güngör et al. [13] (2012)	Diagnostic accuracy after the 30-minute reassessment	Low risk	Low risk	Low risk	Low risk	Low risk	Low risk
Oğuztürk et al. [25] (2012)	Diagnostic accuracy of the early diagnosis following the 20- and 40-minute reassessments compared with final diagnosis	Some concerns – an online block-randomization schedule was used, but allocation concealment before treatment assignment was not clearly described	Low risk	Low risk	Low risk	Low risk	Some concerns
Agodirin et al. [14] (2013)	Change in provisional clinical diagnosis 15–20 minutes after study drug administration	Some concerns – treatment was assigned by patient selection of an envelope from an opaque bag, but generation of the allocation sequence was not clearly described	Low risk	Low risk	Low risk	Low risk	Some concerns
Gülen et al. [26] (2016)	VAS pain relief at 30 minutes	Low risk	Low risk	Low risk	Low risk	Low risk – pain relief at 30 min was explicitly identified as the primary outcome	Low risk
Farnia et al. [27] (2016)	VAS pain score at 30 minutes	Low risk	Low risk	Low risk	Low risk	Low risk – the specified pain assessments were reported without evidence of selective outcome reporting	Low risk
Chinn et al. [28] (2019)	Change in 0–10 pain score from baseline to 90 minutes	Low risk	Low risk	Low risk	Some concerns – patient-reported pain was the primary outcome, and the investigators reported that treatment blinding may have been imperfect	Low risk	Some concerns
Akhgar et al. [29] (2021)	NRS pain reduction at 60 minutes	Low risk	Low risk	Low risk	Low risk	Low risk	Low risk
Priyanka et al. [30] (2025)	NRS pain score at 1 hour	Some concerns – computer-generated randomization was reported, but allocation concealment was not described	Low risk	Low risk	Some concerns – pain was patient-reported in a procedural comparison; although patients and outcome-assessing nurses were described as blinded, maintenance of patient blinding between ESP block and IV tramadol was not clearly demonstrated	Low risk	Some concerns

**Table 3:** Appendix 2: Comprehensive summary of included studies. Scale ranges and units are reported as specified in the individual studies. CI = confidence interval; ED = emergency department; ESP =  erector spinae plane; IM = intramuscular; IV = intravenous; NRS = Numeric Rating Scale; RCT = randomized controlled trial; RR = risk ratio; RUQ = right upper quadrant; SMS = sonographic Murphy sign; VAS = Visual Analog Scale; VRS = verbal rating scale

Study	Study design	Population	Intervention/Comparator	Primary relevant endpoint	Main findings	Adverse events
Pace and Burke [19] (1996)	Prospective, randomized, double-blind, placebo-controlled trial	75 adults with acute atraumatic abdominal pain enrolled; 71 completed the study: 35 morphine and 36 placebo	Titrated IV morphine sulfate, maximum 20 mg, vs. normal-saline placebo	Change in VAS pain score and accuracy of diagnostic evaluation	Mean VAS improvement was 3.9 ± 2.8 cm with morphine vs. 0.8 ± 1.5 cm with placebo (p < 0.01). There was no significant difference in diagnostic accuracy, and three diagnostic or management errors occurred in each group	Side effects were minimal; nausea occurred in 2 morphine patients and 1 placebo patient
LoVecchio et al. [12] (1997)	Prospective, randomized, placebo-controlled trial	49 adults with acute abdominal pain and peritoneal findings enrolled; 48 completed the study	Morphine 10 mg IV vs. morphine 5 mg IV vs. placebo	Change in physical examination findings following analgesia	Pain decreased significantly in both morphine groups. Significant changes in tenderness or localization occurred in the morphine groups but not the placebo group. No delay in diagnosis or morbidity was attributed to analgesia	No adverse effects from the study infusion were reported
Dula et al. [20] (2001)	Prospective, randomized, double-blind trial	30 adults with confirmed acute biliary colic; 16 ketorolac and 14 meperidine	Ketorolac 60 mg IM vs. meperidine 1.5 mg/kg IM, maximum 100 mg	Pain relief at 30 minutes	Mean pain reduction on a 1–10 VAS was 3.8 with ketorolac vs. 3.9 with meperidine (p = 0.962). Rescue analgesia was required in 2/16 (12.5%) vs. 4/14 (28.6%), respectively	Adverse events were not a principal reported outcome
Henderson et al. [21] (2002)	Prospective, randomized, double-blind trial	324 adults with biliary colic completed the protocol; 175 ketorolac and 149 meperidine	Ketorolac 30 mg IV vs. meperidine 50 mg IV	Pain relief using 0–10 VAS and 4-point VRS through 2 hours	No significant difference in pain control was found at any measured interval. Mean VAS improvement at 2 hours was 6.2 ± 3.6 cm with ketorolac vs. 6.7 ± 3.6 cm with meperidine (p = 0.25)	Nausea occurred in 6.8% vs. 16.4%, dizziness in 6.8% vs. 17.9%, and drowsiness in 34.6% vs. 42.1%, respectively
Thomas et al. [9] (2003)	Prospective, randomized, double-blind, placebo-controlled trial	74 adults with undifferentiated acute abdominal pain	Titrated IV morphine sulfate vs. saline placebo	Effect of morphine on physical examination findings and diagnostic accuracy	Diagnostic accuracy was 64.2% with morphine vs. 66.7% with placebo (RR = 0.96, 95% CI = 0.73–1.27). There was no evidence that morphine masked clinically important physical examination findings	No study-medication side effects were reported
Gallagher et al. [22] (2006)	Randomized, double-blind, placebo-controlled trial	160 adults randomized; 153 available for analysis, 78 morphine and 75 placebo	Morphine 0.1 mg/kg IV vs. placebo	Clinically important diagnostic accuracy after analgesia	Median VAS reduction at 15 minutes was 33 mm with morphine vs. 2 mm with placebo. Diagnostic accuracy was 86% vs. 85%; difference 1% (95% CI = -11% to 12%)	No patient required naloxone
Olsen et al. [23] (2008)	Prospective, randomized, double-blind trial	46 adults with suspected biliary colic; 21 ketorolac and 25 butorphanol	Ketorolac 30 mg IV vs. butorphanol 1 mg IV	Pain relief at 15 and 30 minutes	Pain scores favored butorphanol at 15 minutes (p = 0.047), while the between-group difference was not significant at 30 minutes (p = 0.45). Rescue analgesia was required in 6/21 (29%) vs. 5/25 (20%), respectively (p = 0.50)	Nausea occurred more frequently with ketorolac (24% vs. 4%); sedation (5% vs. 36%) and dizziness (0% vs. 28%) occurred more frequently with butorphanol
Noble et al. [24] (2010)	Prospective randomized placebo-controlled study	30 ED patients with abdominal pain undergoing right-upper-quadrant ultrasound; 15 meperidine and 15 placebo	Meperidine 5 mg vs. placebo before repeat ultrasound evaluation	Effect of analgesia on the sonographic Murphy sign	Only 1/30 patients had a change in the emergency physician-assessed sonographic Murphy sign, and that patient received placebo. Post-medication assessment had sensitivity 0.82 (95% CI = 0.48–0.97) and specificity 0.95 (95% CI = 0.71–0.99) for cholecystitis	Adverse events were not a principal reported outcome
Güngör et al. [13] (2012)	Randomized, blinded, placebo-controlled trial	80 adults aged ≥65 years with undifferentiated acute abdominal pain; 39 morphine and 41 placebo	Morphine 0.1 mg/kg IV vs. saline placebo	Clinically important change in diagnostic accuracy and physical examination	Diagnostic accuracy was 80% with morphine vs. 78% with placebo; difference 2% (95% CI = -7% to 13%; p = 0.9802). Abdominal rigidity changed significantly more often after morphine	Overall adverse events occurred in 11/39 (28%) vs. 5/41 (12%); difference 16% (95% CI = -4% to 36%; p = 0.10)
Oğuztürk et al. [25] (2012)	Prospective, randomized, double-blind, placebo-controlled trial	210 adults with nontraumatic acute abdominal pain; 70 per group	Tramadol 1 mg/kg IV vs. paracetamol 15 mg/kg IV vs. placebo	VAS pain reduction, abdominal examination findings, and diagnostic accuracy at 20 and 40 minutes	Median VAS decreased from 85 to 27.5 with tramadol and 83 to 33 with paracetamol at 40 minutes, compared with 83.5 to 85 with placebo. Diagnostic accuracy was 96%, 94%, and 94%, respectively	New or worsening nausea/vomiting occurred in 14/70 tramadol and 13/70 paracetamol patients; 1 tramadol patient reported dizziness. No patient required intervention for an adverse effect
Agodirin et al. [14] (2013)	Prospective, randomized, double-blind, placebo-controlled trial	95 adults aged 18–60 years with nontraumatic acute abdomen; 46 tramadol and 49 placebo	Tramadol 100 mg IV vs. water-for-injection placebo	Changes in pain, physical findings, provisional diagnosis, and treatment decisions	Pain improvement was greater with tramadol (p = 0.001), with improved abdominal palpation findings (p = 0.02). Diagnosis changed in 10/46 tramadol patients vs. 2/49 placebo patients (p = 0.01); 8 of the 10 changes after tramadol moved toward the final diagnosis	Complications or new symptoms occurred in 5/46 tramadol patients and 2/49 placebo patients (p = 0.19). All reported events were non-severe, and no patient developed respiratory difficulty or hypotension
Gülen et al. [26] (2016)	Prospective, randomized, single-blind controlled trial	90 adults with acute pancreatitis; 30 patients per group	Paracetamol 1 g IV vs. dexketoprofen 50 mg IV vs. tramadol 1 mg/kg IV	Pain relief at 30 minutes measured using a 100-mm VAS	Median VAS reductions were 41.5 with paracetamol, 40.5 with dexketoprofen, and 45.5 with tramadol; there was no significant between-group difference (p = 0.38). Rescue morphine was required in 4/30 (13.3%), 6/30 (20%), and 3/30 (10%), respectively	Nausea/vomiting occurred in 1 paracetamol patient, 2 dexketoprofen patients, and 2 tramadol patients; 1 brief hypotensive episode occurred with tramadol
Farnia et al. [27] (2016)	Double-blind randomized controlled trial	87 adults with confirmed biliary colic; 44 combination therapy and 43 standard-dose morphine	Morphine 0.05 mg/kg IV + paracetamol 1 g IV vs. morphine 0.1 mg/kg IV + saline placebo	VAS pain scores at 15 and 30 minutes	Mean VAS was 2.16 vs. 2.51 at 15 minutes; difference -0.35 (95% CI = -1.15 to 0.45; p = 0.38) and 1.66 vs. 2.14 at 30 minutes; difference -0.48 (95% CI = -1.20 to 0.24; p = 0.19)	Nausea: 3/44 vs. 4/43; vomiting: 2/44 vs. 1/43; sedation: 3/44 vs. 1/43; dizziness: 2/44 vs. 1/43. No hypotension, allergic reactions, or respiratory depression occurred
Chinn et al. [28] (2019)	Double-blind randomized controlled trial	154 adults with acute abdominal pain; 77 per group	Lidocaine 120 mg IV vs. hydromorphone 1 mg IV	Improvement in 0–10 pain score from baseline to 90 minutes	Mean improvement was 3.8 with lidocaine vs. 5.0 with hydromorphone; difference 1.2 points (95% CI = 0.3–2.2). Rescue analgesia was required in 39/77 (51%) vs. 20/77 (26%); difference 25% (95% CI = 10%–40%)	Medication-associated symptoms occurred in 23/77 (30%) vs. 28/77 (36%). No serious adverse events occurred, and no patient required naloxone
Akhgar et al. [29] (2021)	Double-blind randomized clinical trial	104 adults aged 18-64 years with suspected biliary colic; 51 lidocaine and 53 morphine	Lidocaine 100 mg IV vs. morphine sulphate 5 mg IV	NRS pain reduction at 60 minutes; NRS also assessed through 120 minutes	NRS was lower with lidocaine at 10 minutes and lower with morphine at 30 minutes. There was no significant difference at the prespecified 60-minute primary endpoint. Rescue fentanyl was required in 10/51 (19.6%) vs. 4/53 (7.5%), respectively	Dizziness occurred in 5/51 (9.8%) lidocaine patients and vomiting in 4/53 (7.5%) morphine patients. No adverse event required physician intervention
Priyanka et al. [30] (2025)	Single-blind randomized controlled trial	100 adults aged 18-70 years with acute pancreatitis; 50 per group	Bilateral ESP block with ropivacaine 0.375%, 40 mL, vs. tramadol 1 mg/kg IV every 6 hours; fentanyl rescue available in both groups	NRS 0-10 pain scores, hemodynamic parameters, and rescue analgesic requirements	NRS was 1.54 ± 0.67 with ESP block vs. 3.18 ± 0.66 with tramadol at 1 hour (p < 0.001), with lower scores through 16 hours. Rescue fentanyl was required in 7/50 (14%) vs. 47/50 (94%) patients (p < 0.001)	No complications or adverse drug reactions were reported in either group
